# Outcomes comparison of elastic bandage versus lower-leg cast immobilization after anterior talofibular ligament repair

**DOI:** 10.1186/s12891-024-07584-x

**Published:** 2024-06-15

**Authors:** Ziyi Chen, Yujie Zhao, Xiaoao Xue, Xicheng Gu, Yinghui Hua

**Affiliations:** 1 Department of Sports Medicine, Huashan Hospital, Fudan University, Shanghai, China; 2 Department of Nursing, Huashan Hospital, Fudan University, Shanghai, China

**Keywords:** Elastic bandage, Lower-leg cast, Anterior talofibular ligament, Arthroscopic repair

## Abstract

**Purpose:**

The aim of this study was to compare the clinical outcomes between patients with chronic ankle instability (CAI) undergoing arthroscopic anterior talofibular ligament (ATFL) repair who received elastic bandage treatment and those who received lower-leg cast immobilization.

**Methods:**

CAI patients with isolated ATFL injury undergoing arthroscopic ATFL repair from January 2017 and August 2019 were included in the study. The visual analogue scale (VAS) at rest and during activities, American Orthopedic Foot and Ankle Society (AOFAS) score, Karlsson Ankle Functional Score (Karlsson score), and time of returning to walk, walk normally, work and sports were evaluated preoperatively, and at 6 months and 12 months follow-up.

**Results:**

A total of 41 patients were included in this study. Among them, 24 patients accepted lower-leg cast fixation, and the other 17 patients were immobilized with elastic bandage. Compared to patients with lower-leg immobilization, patients with elastic bandage fixation had significantly lower VAS during activities (*P* = 0.021) and higher AOFAS score (*P* = 0.015) at 12 months follow-up. The Karlsson score at 6 months follow-up were significantly higher in elastic bandage group than those in lower-leg group (*P* = 0.011). However, no significant difference was observed in time of returning to walk, work and sports between the two groups.

**Conclusion:**

Elastic bandage treatment was better than lower-leg cast immobilization in terms of eliminating pain symptom at 12 months follow-up, and improving ankle functional outcome at 6 months follow-up. Moreover, the present study emphasized that lower-leg cast immobilization offered no advantages in arthroscopic ATFL repair postoperative immobilization.

**Study design:**

Cohort study; Level of evidence, 3.

**Supplementary Information:**

The online version contains supplementary material available at 10.1186/s12891-024-07584-x.

## Introduction

Ankle sprain is the most common musculoskeletal injuries [1]. It accounts for over 10% of all sports injuries, with an incidence rate of approximately 1 per 1000 exposures [2]. Up to 85% of patients with ankle sprain sustain anterior talofibular ligament (ATFL) injury [3]. Although some patients can be cured after conservative treatment, as many as 40% of patients suffer from persistent symptoms of pain, swelling, or a feeling of instability, and eventually develop chronic ankle instability (CAI) [2]. Surgical intervention is recommended in patients exhibiting symptoms of CAI after 3 to 6 months of nonsurgical treatment [3]. Arthroscopic ATFL repair has been most frequently used for CAI patients because of less post-operative pain and recovery time, superior management for concomitant articular injuries and cosmetic appearance [4].

There are frequently conflicts in choosing immobilization methods after arthroscopic ATFL repair. Some orthopedic surgeons choose to immobilize the ankle with lower-leg cast to protect the surgical repair, while others hold that immobilization of the ankle with elastic bandage allowing for early joint mobility thereby avoiding stiffness and other complications of cast [5–7]. Over the past decades, with the need for early functional rehabilitation and the awareness of issues such as thromboembolic events, neuromuscular deconditioning and chronic regional pain syndrome increasing, the balance has swung toward early postoperative interventions [6]. Current studies also show that postoperative treatments of CAI are as important as surgery, which is beneficial to avoid ankle joint limited range of motion, loss of muscle strength, proprioception and balance, gait coordination and many other problems [6]. 

However, there is no uniform standard method for ankle immobilization after ankle ligament repair, and there is a great deal of variation [5–7]. In this study, we aimed to compare the clinical outcomes between patients with CAI undergoing arthroscopic ATFL repair who received elastic bandage treatment and those who received lower-leg cast immobilization. We hypothesized that elastic bandage treatment offered better functional outcomes than lower-leg cast immobilization, offering earlier functional rehabilitation and more comfortable living experience for patients.

## Materials and methods

### Study design

Consecutive patients who underwent arthroscopic ATFL repair between January 2017 and August 2019 were included in this study. The inclusion criteria were as follows: (1) patients exhibit symptoms of CAI after 3 to 6 months of nonsurgical treatment, (2) physical examination exhibit tenderness around the lateral ligaments or a positive anterior drawer test, (3) a stress radiography or magnetic resonance imaging, and surgery was suggested [3]. Patients with the following conditions were excluded: (1) obvious bone deformity, (2) neuromuscular disorders, (3) general joint laxity, (4) augmented ATFL reconstruction with tendons, or (5) calcaneofibular ligament (CFL) or deltoid ligament tears [7]. 

Patient demographics, medical records, surgical documents and postoperative fixation methods were detailed documented for all patients. All patients were followed up at hospital or by telephone using subjective questionnaires preoperatively, 6 months after surgery (short term follow-up for CAI), and 12 months after surgery (mid-term follow-up for CAI). Clinical outcome measures included the following: visual analogue scale (VAS) at rest and during activities, American Orthopedic Foot and Ankle Society (AOFAS) score, Karlsson Ankle Functional Score (Karlsson score), time of returning to walk and normal walk, time of returning to work, and time of returning to sports.

### Treatment methods

Surgeons performed surgical procedures in accordance with the methods described by Chen et al. [7]. Before performing arthroscopic ATFL repair, patients underwent an arthroscopic evaluation of the ankle joint, and the intra-articular injuries were addressed and recorded.

Ankles were immobilized with either lower-leg casts or elastic bandages in a neutral position postoperatively randomly. The postoperative rehabilitation protocol was guided by a physical therapist after the operation. Rehabilitation exercises, including isometric contraction of muscle groups around the ankle joint, were started from the day after surgery. All the ankles were immobilized for 2 weeks, after which the casts or bandages were removed at a clinic. Passive range of motion was encouraged 2 weeks after surgery, and full weight bearing was permitted after 4 weeks. This treatment protocol was similar to that used in previous reports [5, 7].

### Clinical evaluation

Clinical examination was performed by an experienced nurse who was blinded regarding the postoperative fixation methods. Functional examinations included the pre- and post-operative VAS at rest and during activities, AOFAS score, Karlsson score, time of returning to walk and normal walk, time of returning to work, and time of returning to sports. Preoperative clinical scores were obtained before the patients underwent the arthroscopy surgery, and post-operative scores were acquired 6 months and 12 months after surgery for each patient.

### Statistical analysis

All statistical tests were performed using the IBM SPSS Statistics for Windows version 23.0 (SPSS Inc., IBM, Chicago, IL). The Shapiro–Wilk test and Levene test were used to determine whether the quantitative data conformed to normal distribution and variance chi-square. Normally distributed quantitative data were expressed as mean ± standard deviations, skewed distributed quantitative data were displayed as median (Q1, Q3). In the comparison of participants characteristics and preoperative functionals score between the two groups, the two independent samples t-test was used for normally distributed quantitative variables, the two independent samples Mann-Whitney U rank-sum test for stewed distributed quantitative variables, and the χ² test for categorical variables. Then preoperative function score variables with a significant P value (*P* < 0.1) were entered into a multiple linear regression analysis [7]. A multiple linear regression analysis was conducted with the postoperative functional scores serving as the criterion variables and fixation methods along with preoperative functional scores serving as the explanatory variables. The multiple linear regression model allowed an adjustment for confounding variables such as preoperative functional scores. For time of returning to walk, work and sports between the two groups, the two independent samples Mann-Whitney U rank-sum test were performed. The level of statistically significant difference was set as a *P* < 0.05. Post hoc analysis of difference between two independent means (two groups) was performed to calculate the power of our study. As the calculated effect size, sample size, and α level were 0.8, 24 for group 1 and 17 for group 2, 0.05, respectively, the power was determined to be 0.8.

## Results

A total of 41 patients with CAI were included in this study. Among them, 24 patients were immobilized with lower-leg casts, and the other 17 patients received elastic bandages fixation. Baseline demographic data are presented in Table 1. No significant difference was observed in age, sex, height, weight, body mass index (BMI), side affected, heavy labor, and osteochondral lesions (*P* = 0.295, 0.063, 0.385, 0.141, 0.354, 1.000, 0.450, 0.259). Preoperative functional scores between two groups were demonstrated in Table S1. No patients in either group had ankle instability after surgery. No patients experienced wound infection or numbness in their ankles. No patient in either group underwent revision lateral ligament surgery.

**Table 1:** Participant characteristics. Data are expressed at median (minimum-maximum) or number. BMI, body mass index

	Lower-leg cast (*n* = 24)	Elastic bandage (*n* = 17)	*P* value
Age, years	33.67 ± 9.52	32.37 ± 8.04	0.295
Sex, male/female	11/13	13/4	0.062
Height, m	167.50 ± 8.11	173.47 ± 7.73	0.385
Weight, kg	64.5 (49.5, 78.5)	72 (59, 82.5)	0.141
BMI, kg/m^2^	22.94 (18.21, 24.83)	23.37 (20.41, 26.55)	0.354
Side affected, left/right	15/9	11/6	1.000
Heavy labor, n	4	5	0.450
Osteochondral lesion, n	10	6	0.259

As displayed in Table 2, compared to patients with lower-leg immobilization, patients with elastic bandage fixation had significantly lower VAS during activities (*P* = 0.021) and higher AOFAS score (*P* = 0.015) at 12 months follow-up, while no significant difference were observed at 6 months follow-up (*P* = 0.052, 0.097).

**Table 2:** Multiple linear regression analysis of fixation methods associated with pain evaluated by VAS at rest, VAS during activities, AOFAS score. VAS, visual analogue scale, AOFAS, American Orthopedic Foot and Ankle Society

	VAS at rest				VAS during activities				AOFAS score			
	6 months		12 months		6 months		12 months		6 months		12 months	
	B	P	B	P	B	P	B	P	B	P	B	P
Preoperative score	0.260	0.001	-0.006	0.613	0.277	0.055	0.120	0.345	0.158	0.013	0.083	0.151
**Cast/Elastic bandage**	-0.056	0.860	0.005	0.351	-1.096	0.052	-1.177	0.021*	3.454	0.097	4.811	0.015*

At both 6 months and 12 months follow-up, there were no significant difference in VAS at rest (*P* = 0.860, 0.351). Additionally, there is a significant difference in Karlsson score at 6 months follow-up between lower-leg cast group and elastic bandage group (*P* = 0.011), while no significant difference at 12 months follow-up (*P* = 0.079) (Table 3). As for time of returning to walk, walk normally, work and sports, there was no significant difference between two groups (*P* = 0.154, 0.061, 0.859, 0.865) (Table 4).

**Table 3:** Karlsson score between lower-leg cast and elastic bandage groups. Data are expressed at median (minimum-maximum). * represents *P* < 0.05. Karlsson score, Karlsson Ankle Functional Score

	KAFS			
	6 months		12 months	
	B	P	B	P
Preoperative score	0.221	0.012	6.321	0.013
**Cast/Elastic bandage**	9.877	0.011*	0.208	0.079

**Table 4:** Time of returning to walk, work and sports between patients immobilized with lower-leg cast and elastic bandage. Data are expressed at median (minimum-maximum)

	Lower-leg cast (*n* = 24)	Elastic bandage (*n* = 17)	*P* value
Time of returning to walk, weeks	7 (3.75, 8)	4 (3, 8)	0.154
Time of returning to walk normally, weeks	12 (6, 14.5)	8 (6, 12)	0.061
Time of returning to work, months	3 (2, 3.5)	4 (2, 6)	0.859
Time of returning to sports, months	6 (3.75, 12)	7 (3, 10)	0.865

## Discussion

Our study was the first to discuss the most appropriate immobilization methods after arthroscopic ATFL repair for CAI patients. We found that postoperative elastic bandage treatment was better than lower-leg cast immobilization in terms of eliminating pain symptom at 12 months follow-up, and improving ankle functional outcome at 6 months follow-up. Moreover, the present study emphasized that lower-leg cast immobilization offered no advantages in arthroscopic ATFL repair postoperative immobilization.

Arthroscopic ATFL repair is widely accepted as the reference standard for the surgical managements of CAI patients, allowing immediate weightbearing and returning high-demand athletes to their preinjury sports level [3, 4, 8]. However, choosing the appropriate postoperative ankle fixation method has always been a highly controversial issue. Some orthopedic surgeons suggested that the ideal rehabilitation regime after ATFL repair surgery was lower-leg cast for 1 or 2 weeks, followed by 2–4 weeks in a walking boot [9], whereas other people revealed that a compressive bandage and walker boot in neutral position were used for the first 2 weeks with no weight bearing [10]. Serkan et al. reported that elastic bandage treatment was superior to cast immobilization in terms of preserving ankle muscle strength, clinical outcomes, and functional scores in treating tuberosity fractures [11]. However, similar studies in CAI patients after arthroscopic ATFL repair are yet to be conducted. Most studies have focused on the external supports for acute ankle sprain [12–14]. 

Our results demonstrated that compared to patients with lower-leg immobilization, patients with elastic bandage fixation had significantly lower VAS during activities and higher AOFAS score at 12 months follow-up, while no significant difference were observed at 6 months follow-up. The VAS is one of the most reliable and valid measurement tools for self-report of pain [15]. They are typically presented as a 10 cm line with descriptive anchors at each end, such as “completed all prescribed activities today” to “completed none of the prescribed activities today.” [15]. The AOFAS is one scoring system used to assess and monitor the progress of patients after foot and ankle surgery, with 40% scores indicates pain symptom [16]. Similarly, Bayram et al. found that elastic bandage treatment was better than cast immobilization in terms of alleviating pain symptoms [11]. Akimau et al. compared elasticated bandages and short leg casts using VAS scoring, subsequently showing no significant differences between the 2 treatment methods [17]. Our research indicated that elastic bandage fixation after arthroscopic ATFL repair surgery might reduce patients’ pain experience in the long term, which would improve postoperative patients’ life quality to some extent. Karlsson score is a relatively more comprehensive scoring scale for evaluation of ankle joint function, overlapping pain, swelling, instability, stiffness, stair climbing, running, work activities and support—8 parts of ankle function [18]. Bayram et al. reported elastic bandage treatment was better than cast immobilization in terms of preserving ankle muscle strength, clinical outcomes, and functional scores [11]. Our study found that elastic bandage group had a significant higher Karlsson score at 6 months follow-up than lower-leg cast group and, while no significant difference at 12 months follow-up. It suggested that immobilization with elastic bandage which allowed joint mobility might facilitate early functional rehabilitation. We also discovered that lower-leg cast immobilization after arthroscopic ATFL repair offered no advantages in returning to walk, work and sports. Additionally, when using casts, patients often experienced discomfort, inconvenience in life, and might develop potential complications such as thromboembolic events, neuromuscular deconditioning and chronic regional pain syndrome [19]. Overall, we believed that the use of elastic bandages immobilization after arthroscopic ATFL repair is a more idea postoperative rehabilitation option.

Therefore, for CAI patients without concomitant injuries such as CFL injury, osteochondral injury and so on after arthroscopic ATFL repair, elastic bandage fixation is a good choice after full evaluation by doctors. Specifically, in clinical practice, elastic bandage fixation should be preferred for patients with chronic regional pain syndrome, sports athletes, and other patients who have a strong desire to restore ankle function as soon as possible, and those who believe that plaster discomfort affects normal life. Some limitations still reside in this research. First, this study is a retrospective cohort study with a relatively small sample size, so there is a large chance to generate false positive results. Prospective, long-term comparative and randomized studies with larger sample size are needed to verify whether elastic bandage is superior to lower-leg cast fixation after arthroscopic ATFL repair. Moreover, patients included in this study only had isolated ATFL tear. More researches should be conducted to discuss whether elastic bandage immobilization is still superior to lower-leg cast in patients with multiple ligament lesions such as CFL or deltoid ligament injuries. Last, we didn’t make a comparison of the mechanical and structural quality of the tissues in cast and elastic bandage group, and further study could be conducted in the future.

## Conclusion

Our study was the first study to evaluate the outcomes of elastic bandage versus lower-leg cast immobilization after arthroscopic ATFL repair. Elastic bandage treatment was better than lower-leg cast immobilization in terms of eliminating pain symptom at 12 months follow-up, and improving ankle functional outcome at 6 months follow-up. Moreover, the present study emphasized that lower-leg cast immobilization after arthroscopic ATFL repair offered no advantages in returning to walk, work and sports.

### Electronic supplementary material

Below is the link to the electronic supplementary material.

Supplementary Material 1

## Data Availability

The datasets used and/or analyzed during the current study available from the corresponding author on reasonable request.
