# Supplementary material for: Outcomes comparison of elastic bandage versus lower-leg cast immobilization after anterior talofibular ligament repair
Source: BMC Musculoskelet Disord. 2024 Jun 15;25:469. doi: 10.1186/s12891-024-07584-x (PMC11179373; doi:10.1186/s12891-024-07584-x)
Supplement: Supplementary file 1 — Supplementary Material 1 [file 12891_2024_7584_MOESM1_ESM.docx]

|  |  | **Lower-leg cast**  **(n=24)** | **Elastic bandage (n=17)** | **P value** |
| --- | --- | --- | --- | --- |
|  | VAS at rest | 2 (1.5, 4) | 0 (0, 2) | **0.022*** |
|  | VAS during activities | 6.46±1.62 | 4.56±1.87 | **0.001**** |
|  | AOFAS score | 52.83±15.85 | 66.53±14.68 | **0.008**** |
|  | KAFS | 23.50 (20, 37.75) | 27 (16, 67) | 0.085 |
|  | **Table S1:** Preoperative function score. Data are expressed at median (minimum-maximum). * represents P < 0.05 and ** represents P < 0.01. VAS, visual analogue scale; AOFAS American Orthopedic Foot and Ankle Society, KAFS Karlsson Ankle Functional Score. | | | |
